# Engaging students through online video homework assignments: A case study in a large‐enrollment ecology and evolution course

**DOI:** 10.1002/ece3.7547

**Published:** 2021-05-01

**Authors:** Laci M. Gerhart, Brittany N. Anderton

**Affiliations:** ^1^ University of California, Davis Davis CA USA; ^2^ iBiology San Francisco CA USA

**Keywords:** ecology, evolution, online learning, online videos, video homework, YouTube videos

## Abstract

Online educational videos have the potential to enhance undergraduate biology learning, for example by showcasing contemporary scientific research and providing content coverage. Here, we describe the integration of nine videos into a large‐enrollment (*n* = 356) introductory evolution and ecology course via weekly homework assignments. We predicted that videos that feature research stories from contemporary scientists could reinforce topics introduced in lecture and provide students with novel insights into the nature of scientific research. Using qualitative analysis of open‐ended written feedback from the students on each video assigned throughout the term (*n* = 133–229 responses per video) and on end‐of‐quarter evaluations (*n* = 243), we identified common categories of student perspectives. All videos received more positive than negative comments and all videos received comments indicating that students found them intellectually and emotionally stimulating, accessible, and relevant to course content. Additionally, all videos also received comments indicating some students found them intellectually unstimulating, though these comments were generally far less numerous than positive comments. Students responded positively to videos that incorporated at least one of the following: documentary‐style filming, very clear links to course content (especially hands‐on activities completed by the students), relevance to recent world events, clarity on difficult topics, and/or charismatic narrators or species. We discuss opportunities and challenges for the use of online educational videos in teaching ecology and evolution, and we provide guidelines instructors can use to integrate them into their courses.

## Graphical abstract

In this case study, we sought to evaluate student perceptions of freely available educational videos and how such videos may contribute to student learning goals. We report the use of nine online videos for teaching concepts and competencies in evolution and ecology in a large‐enrollment introductory biology course. We share the results of our case study and provide guidance for the use of online videos in teaching evolution and ecology in higher education.

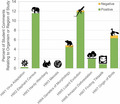

## 1 INTRODUCTION

Video has been used in education for decades, and there are expanding audiovisual resources for higher education (Betrancourt & Benetos, 2018; Brame, 2016; Duffy, 2008; Moussiades et al., 2019). These resources have become essential during the COVID‐19 pandemic, as educators worldwide are forced to move their courses online with little preparation. In particular, free online videos—such as those on YouTube, or from online education platforms such as iBiology or HHMI Biointeractive—provide biology educators with a wealth of curated educational content that is easy to share with their students remotely.

Unlike lecture capture, which records only the instructor's presentation of material, online videos from external resources can provide unique perspectives on topics and concepts, and they can also showcase broad representations of scientists and their research (Schinske et al., 2016). In addition, videos are amenable to a flipped approach, which frees up valuable synchronous class time for discussion and feedback between educators and students (Bishop & Verleger, 2013; Gross et al., 2015; Herreid & Schiller, 2013; Sletten, 2017). The use of online videos in STEM higher education has been described previously (Barry et al., 2016; Cox, 2011; Dupuis et al., 2013; Rajan & Veguilla, 2018; Schinske et al., 2016) and at least one study reports an association between online videos and higher examination scores, particularly for students with lower grade point averages (Dupuis et al., 2013). Outside of the formal education context, YouTube videos have been shown to promote knowledge acquisition of science content (Boy et al., 2020). To our knowledge, the use of online videos for teaching undergraduate ecology and evolution, and the student perspective on engagement with videos in this context has not been reported.

While this paper discusses the use of video homework assignments in a traditionally structured in‐person course, many educators forced online by the COVID‐19 pandemic are exploring video‐based techniques for remote instruction and may be unable to develop original content for their courses. Now more than ever, educators are likely searching for existing content (e.g., on YouTube) to share with their students online to support or replace instructor‐generated videos. It is important that educators identify quality video content that can support their student learning goals and which students will find engaging. In this case study, we report the use of nine free online educational videos for teaching concepts and competencies in evolution and ecology in a large‐enrollment introductory biology course. The videos were integrated into the curriculum via weekly homework assignments in a traditional (in‐person) course. We collected student feedback on each video and performed a qualitative analysis to identify the strengths and opportunities of this approach. We share the results of our case study and provide guidance for the use of online videos in teaching evolution and ecology.

## 2 MATERIALS & METHODS

### 2.1 Study design

We consider this report a “case study” based on the definition by Case and Light (2011). According to this definition, case studies focus on “a distinct, single instance of a class of phenomena such as an event, an individual, a group, an activity, or a community” (in this case, a single offering of an introductory evolution and ecology course) and are “concerned with the specific application of initiatives or innovations to improve or enhance learning and teaching” (in this case, the use of video‐based homework assignments to promote learning ecology and evolution). According to this definition, case studies are less generalizable than other study structures such as quasi‐experimental or randomized controlled studies. Therefore, we are careful not to generalize our findings beyond the specific context of the course described below.

### 2.2 Course overview

This study was performed in one section of a large‐enrollment (1,300 students per quarter) gateway major introductory biology course focused on evolutionary and ecological concepts at a large research university in the western United States. The course is the first in the introductory series and is a prerequisite for a large number of upper division courses in a wide variety of STEM majors. Enrollment is split across 2–3 sections, ranging in size from 200 to 500 students. The course consists of the following weekly meetings: three 50‐min lectures (full enrollment), one 50‐min “discussion” (full enrollment, often treated as a fourth lecture), and one 180‐min laboratory (24 students per lab section). The campus is a doctoral university with very high research productivity and is a land‐grant, Hispanic‐serving institution of over 30,000 undergraduate students. The academic year is structured into three 10‐week sessions. The course covers introductory content in evolution and ecology, including concepts relating to species interactions, functional diversity, population genetics, and natural selection, as well as skill‐based content such as hypothesis testing and data interpretation. It meets the campus literacy requirements for instruction in visual literacy, scientific literacy, and quantitative literacy as well as the Science and Engineering general education credit.

The learning objectives for the course are:

1. Explain what climate is, what causes it, how it is changing, and how it influences the distribution and abundance of organisms.
2. Explain what biodiversity is and describe how it is measured.
3. Predict how human activities such as overharvesting, habitat destruction, and pollution will affect the diversity and composition of ecological communities and the evolutionary trajectory of species.
4. Describe the concept of a tradeoff and give examples, explaining how specific trade‐offs relate to the maintenance of species diversity in nature.
5. Use the fundamental principles of inheritance to explain the relationship between genotype and phenotype in parents and offspring.
6. Distinguish the processes that lead to limited and unlimited population growth and give examples of factors that limit growth for natural populations.
7. Predict the direction, magnitude, and outcomes of natural selection given a set of biological starting conditions.
8. Describe the contributions that different forms of natural and other forms of selection and genetic drift make to evolutionary change.
9. Use data from population genetics, natural selection, biogeography, and phylogenetics to explain how new species arise.
10. Explain how competition, predation, and mutualism each influence the distribution and abundance of species over time and space.
11. Develop a conceptual framework for global carbon cycling that integrates photosynthesis, primary production, herbivory, decomposition, and the burning of fossil fuels.
12. Interpret graphs and data to evaluate scientific hypotheses, models, and theory for any of the content‐based objectives above (1–11).

Historically, the discussion sessions have been treated as an additional lecture and do not differ from the structure and style of the formal lecture sessions. They include the full course enrollment and so are not a true discussion format (e.g., as described in White & Kolber, 1978). The lead author (LMG) joined the course instructional team in the fall of 2017 and has taught the course 7 times to over 3,100 students. In 2018, LMG instituted a course redesign in which the discussion sessions transitioned into flipped case studies of campus faculty research relevant to the course. The goals of the redesigned discussion sessions were to 1) practice the scientific method (e.g., predicting/interpreting results and accepting/rejecting hypotheses); 2) illustrate current and ongoing research, and 3) introduce students to active research programs on campus relevant to course content, with a focus on diverse representation of scientists*. The redesigned discussion sessions include weekly homework assignments intended to introduce students to how scientists study the topic to be discussed that week. The homework assignments also serve to restructure point values for the course to include more low‐stakes formative assessments, which has been shown to reduce or remove achievement gaps in introductory biology courses (Eddy & Hogan, 2014; Haak et al., 2011).

This course was chosen as a case study for several reasons. First, the course has high enrollment, which is beneficial for generating a large number of responses, and means that the course demographics are similar to campus‐wide demographics (Table 1), both of which reduce potential sources of bias in student responses. Second, it is the first course students take in their major requirements and so serves as a gateway to the rest of the students’ degree. Consequently, students enter the course with a wide range of past experience in biology education and preparedness for college instruction. Additionally, success in the course is critical for preparing students for subsequent major courses and for maintaining timely progress toward degree completion.

**TABLE 1:** Demographic information of course

Demographic	Course Enrollment (total enrollment =356)	Full Campus Enrollment
First Quarter Freshmen	41%	‐‐‐
Females^a^	73%	62%
First Generation (FG)	46.9%	41%
Transfer^b^	8.1%	22%
Low Income (LI)	28.7%	27%
Under‐Represented Minority (URM)	28.7%	28%
International	11.2%	16%
English Language Learners	30.6%	31%
Students Repeating the Course	3.7%	‐‐‐
Academically Distressed^c^	3.7%	6%

^a^ Students may decline to state their gender or may specify a nonbinary gender. This row reflects a rounded percentage of female students.

^b^ Students in the College of Biosciences must transfer to institution with the introductory biology series already completed, which reduces the fraction of transfer students in this course

^c^ This row shows the number of students who were in some form of academic distress in the last quarter they were enrolled. Here academic distress means that the student was on probation, dismissed, subject to dismissal, or continuing probation.

*Both the video homeworks described in this case study and the discussion session activities highlighted individual scientists’ research programs. The discussion session activities were structured around campus researchers whose work related to course content. Diverse representation was more pronounced in the discussion activity scientist profiles than in the video homework speakers.

### 2.3 Structure and goals of the homework assignments

The weekly homework assignments acted as a precursor to the weekly discussion activity. Homework assignments were intended to prepare students for the discussion activities by reinforcing concepts covered in class, linking course topics, refreshing foundational concepts covered in previous biology courses and/or illustrating research applications of course topics. While the video homeworks and in‐class discussion activities focused on different scientists and different research programs, they highlighted research on the same general topic (e.g., trade‐offs). Prior to the term included in this study, the homework assignments consisted of multiple choice quizzes on course topics but did not include online videos.

In the fall 2019 term, students enrolled in the course (*n* = 356) were given weekly homework assignments consisting of viewing one or two online educational videos and responding to 7–8 multiple choice questions relating to the video(s) which were accessed through the course learning management system (LMS). Table 1 summarizes the demographics of students enrolled in the course while Table 2 summarizes the online videos used in the homework assignments. Videos were selected from three common open‐access sources for biology education: iBiology, HHMI BioInteractive, and Bozeman Science. Because this was the first term in which online video was incorporated into the weekly homework assignments for the course, we sought to understand the student perspective on the use of videos for learning ecology and evolution. To that end, open‐ended student feedback on the homework assignments was gathered in two ways:

● Each homework assignment included the following nonanonymous, voluntary, ungraded, open‐ended question: *In the space below, please provide any feedback you have on the video [title]. The more specific you can be in your comments, the more helpful they will be for us. While your answer is not anonymous, I value your honest feedback and both I and the creators of the videos will use your comments to improve the quality of educational videos produced and their integration into courses like [name of course]*.
● The anonymous end‐of‐quarter course evaluations included the following voluntary, open‐ended question: *The video homework assignments were a new component of the course this quarter. Please comment on your opinion of these assignments (whether positive or negative), being as specific as possible*.

**TABLE 2:** Summary of videos used in weekly homework assignments

Assignment	Video Title (Source)	Reasons Videos Were Chosen				Topics Covered
		Topic Refresher	Diving Deeper	Linking Concepts	Research Application	
HW1	Virus Adaptation to Environmental Change (iBiology)		X		X	Trade‐offs
HW2	The Great Elephant Census (HHMI Biointeractive)				X	Census of wild populations
HW3	Youreka Science: Hardy–Weinberg equilibrium (iBiology)	X	X			Allele/genotype frequency calculations
HW3	Phases of Meiosis (Bozeman Science)	X				Process of meiosis
HW4	Genetics of Morphology (iBiology)			X	X	Mechanisms of genetic change
HW5	The Origins of Species: Lizards in an Evolutionary Tree (HHMI Biointeractive)			X	X	Reproductive isolation and speciation
HW6	Consequences of Amazon Deforestation (iBiology)		X		X	Biogeochemical cycling
HW7	Finding *Tiktaalik*(iBiology)		X		X	History of life
HW7	Great Transitions: The Origin of Birds (HHMI Biointeractive)		X		X	History of life

### 2.4 Description of coding methods

Coding was completed using a grounded theory approach (Strauss & Corbin, 1990), similar to that described previously (Seidel et al., 2015). The goal of this approach was to identify and determine the prevalence of major categories of student comments about video homework assignments. First, both authors (LGB and BNA) reviewed a subset of student comments and collaboratively discussed and identified initial categories reflecting the actual language and phrasing used by the students (Saldana, 2015). These categories were refined through an iterative process that included new subsets of student comments and discussion between both coders. Once stable categories were identified, a final codebook including categories and their descriptions was developed (Table 3).

**TABLE 3:** Coding schema for open‐ended student feedback on video homework assignments

Parent Code	Child Code	Description
Usage	Used Transcript or Captions	Explicitly mentioned use of transcripts or captions.
	Desired Transcript or Captions	Explicitly requested access to transcripts or captions.
	Watched multiple times	Included watching multiple times due to video difficulty or due to engagement with an interest in the video topic
	Watched Faster (>1 speed)	Explicitly stated watching the video at a faster speed.
	Watched Slower (<1 speed)	Explicitly stated watching the video at a slower speed.
	Technical Issues	Reported technical problems in video access, viewability, etc.
Positive Comments	General Positive Statement	Unspecific positive statements such as liked/loved the video, or the video was good/great without any clarifying detail.
	Intellectually Stimulating	Including specific phrases such as interesting, intriguing, challenging, informative, educational, helpful, useful, or explicit statement of learning.
	Emotionally Stimulating	Including specific phrases such as engaging, enjoyable, or fun.
	Accessible	Including specific phrases such as clear, easy to understand, straightforward, or well explained.
	Saw Link to Course Content	Explicitly referenced content covered in lecture or laboratory activities.
	Saw Link to Non‐Course Events	Explicitly referenced world events or issues outside of the university setting.
	Process of Science	Including reference to how science works, real studies, experiments and hypothesis testing, scientists in the real world, or real data and graphs.
	Good Length	Explicitly stated that the video was not too long, or was a reasonable length.
	Speaker/Video Style	Including reference to video esthetics (graphics, recording style, etc) and speaker style (enthusiasm, comfort with speaking).
	**Organism or Region of Study**	Including any positive comment specific to the taxonomic group or habitat discussed in the video.
	**Homework Questions**	Including any specific reference to the questions that accompanied the videos.
	*Good Preparation/Study*	Explicitly stating that the homework assignments prepared a student for other coursework such as midterms, or final examinations.
	*Appropriately Difficult*	Explicitly stating that the homework assignments were not unreasonably difficult; including phrases such as challenging in a good way, not too hard, and easy.
	*General Assignment Structure*	Including comments on the format of the assignments and the role they played in the total course grade.
Negative Comments	General Negative Statement	Unspecific negative statements such as disliking the video, or the video were bad, without any clarifying detail.
	Intellectually Unstimulating	Including specific phrases such as hard to follow, difficult to understand, unclear, confusing, dense, overwhelming, “above my level,” or needed more explanation.
	Emotionally Unstimulating	Including specific phrases such as boring, dry, or dull.
	Inaccessible Language	Including specific reference to jargon, terminology, language, or vocabulary.
	Too Long	Including specific phrases such as too long or too time‐consuming.
	Irrelevant	Including specific phrases such as scope too narrow or did not relate to class or life.
	Speaker/Video Style	Including reference to video esthetics (graphics, recording style, etc) and speaker style (awkward, monotone, speaking too fast/slow).
	Homework Questions	Including any specific reference to the questions that accompanied the videos.
	**Organism or Region of Study**	Including any negative comment specific to the taxonomic group or habitat discussed in the video.
	*General Assignment Structure*	Including comments on the format of the assignments and the role they played in the total course grade.

Note:

Codes in bold were used only in individual homework feedback. Codes in italics were used only in end‐of‐quarter feedback

To determine the reliability of the stable categories, the following interrater reliability analysis was performed. A random number generator was used to select a subset of 10% (*n* = 123) of student comments on the homework assignments. The authors separately coded the random subset of student comments using the stable categories. Interrater reliability was measured by percent agreement, and Cohen's Kappa for all codes that were assigned in the random subset of comments. Average percent agreement between coders across all codes assigned in the random subset was 98.07%. Average Cohen's Kappa for all codes assigned in the random subset was 0.82. These values indicate “strong” agreement between the coders (McHugh, 2012).

Once reliability of the codebook was established, student comments on individual videos were coded by a single coder (LMG). In addition to the specific categories described in the codebook (Table 3), each comment was also assigned an Overall Attitude code. Overall Attitude codes included positive, negative, ambivalent, and irrelevant. A comment could be categorized as both positive and negative if it included specific categories of both types (Table 3). Comments were marked as ambivalent only if they included statements such as “It was ok” or “The video was fine” and were not otherwise assigned to specific categories. Comments were marked as irrelevant if they contained comments not pertaining to the assignment or video (e.g., questions or comments about other aspects of the class, “NA”) and thus could not be assigned to specific categories. Usage categories largely related to the speed at which the video was watched, how many times the video was watched, whether a transcript or captions were utilized. While these comments were often associated with positive or negative statements (eg, “It was really confusing, I wouldn't have understood all the jargon without the captions.”) they are not themselves positive or negative and so were coded separately.

Student comments on the video homework structure were also solicited on the anonymous end‐of‐quarter course evaluations. These comments were coded in a similar manner as the individual video comments, including both specific and Overall Attitude codes. Since the nature of the comments differed slightly, the specific categories also differed slightly, though many were similar to those for the individual video comments (Table 3).

### 2.5 IRB/Campus review statement

This case study was determined to be exempt from the UC Davis Institutional Review Board review process based on U.S. Department of Health and Human Services guidelines. It falls under the Quality Assurance/Quality Improvement activities exception as it pertains to assessing or improving a program (in the case, the course) and is not experimental in nature. Additionally, the manuscript was reviewed by representatives of the following campus offices to ensure compliance with student data and privacy guidelines: Center for Educational Effectiveness, Office of Information and Educational Technology, and the University Registrar (the campus FERPA officer).

## 3 RESULTS

### 3.1 Student feedback on individual homework assignments

**FIGURE 1:**
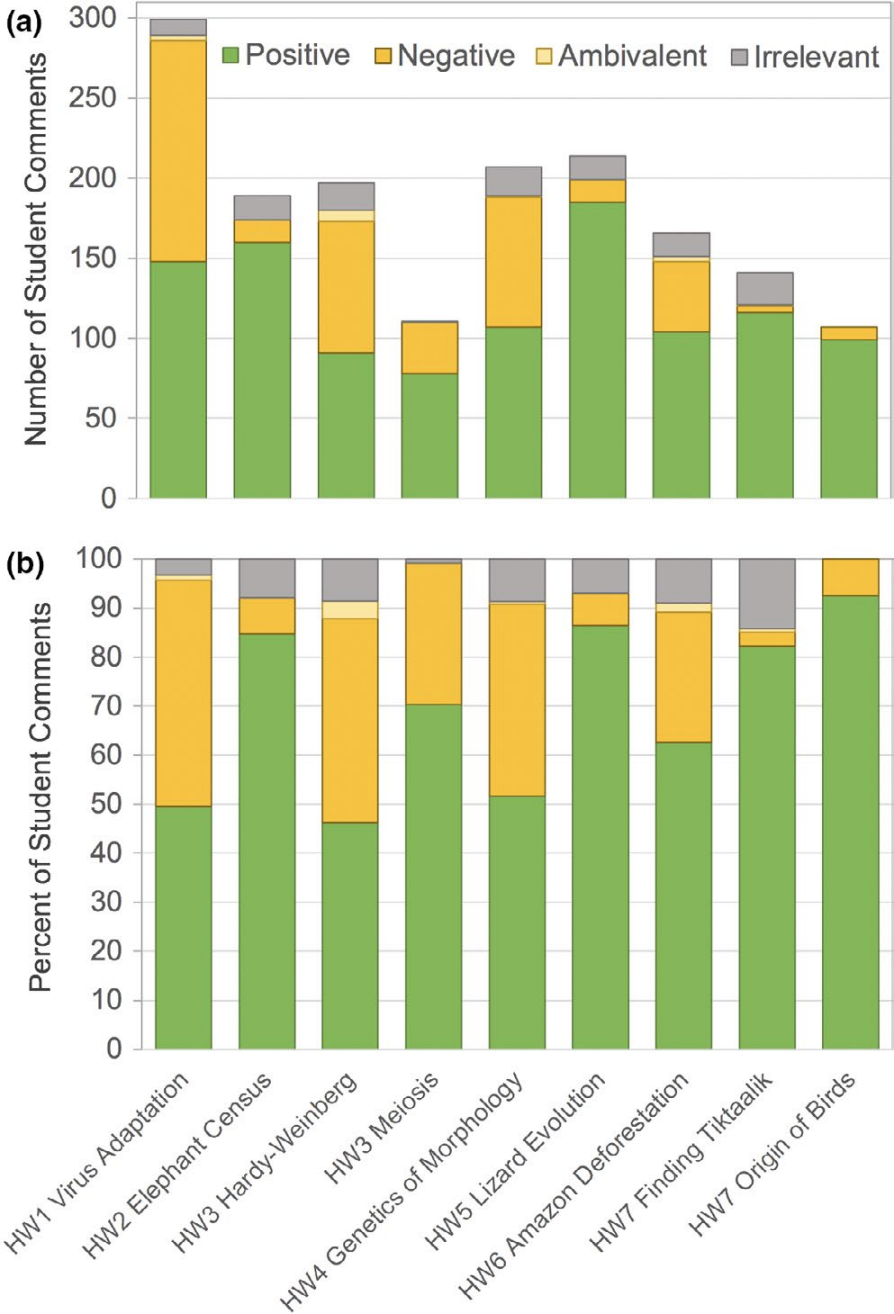
Student attitudes toward nine online educational videos represented by absolute (a) and relative (b) numbers of comment types for each video. Comment types include positive (green), negative (orange), ambivalent (yellow), and irrelevant (gray) overall comments

**FIGURE 2:**
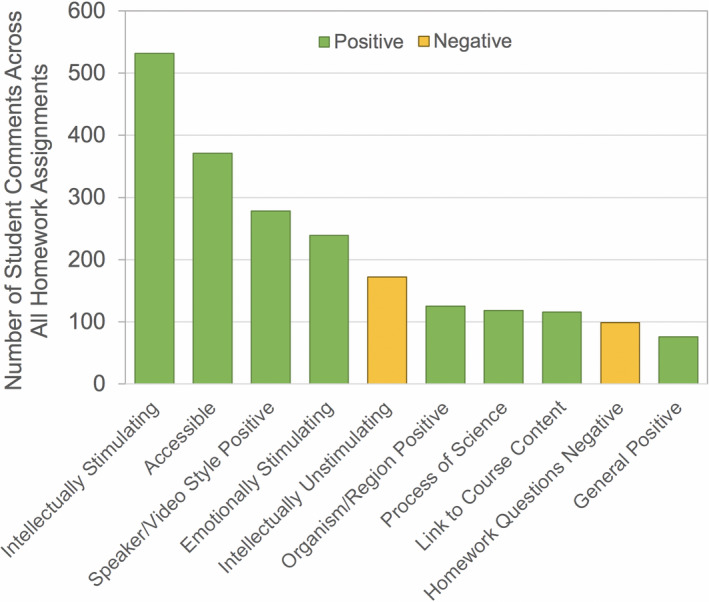
The 10 most common categories identified in student feedback on individual videos, including positive (green) and negative (orange) responses

Open‐ended feedback on the homework assignments was voluntary and solicited anywhere from 133 to 229 individual responses representing 37%–64% of the enrolled students (Table 4). All videos received more positive than negative comments (Figure 1). The number of comments varied widely between videos and generally declined throughout the quarter (Figure 1a). Across all homework assignments, the top 10 most common responses included eight positive categories and two negative categories (Figure 2). Interestingly, one of the negative categories related not to the videos themselves, but to the homework questions developed in association with the videos. All other top categories reflected aspects of the video content or style.

**TABLE 4:** Counts of student responses, per homework assignment

Assignment	Number of Responses	Percent of class (total enrollment =356)
Homework 1	229	64
Homework 2	182	51
Homework 3	174	49
Homework 4	162	46
Homework 5	205	58
Homework 6	133	37
Homework 7	138	39
End‐of‐quarter course evaluations	243	68

#### 3.1.1 Comments on usage and technical issues

**FIGURE 3:**
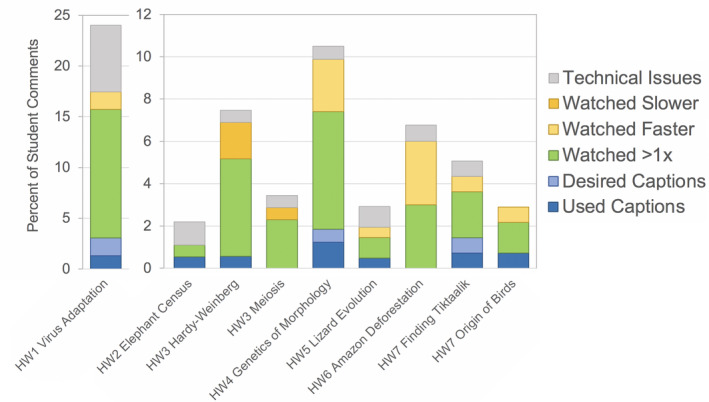
Comments on usage and technical issues by video, as reported in student comments. *Note the difference in y‐axis scales for Homework 1* *versus Homeworks 2–7*

With the exception of Homework 1, comments on usage and technical issues were relatively uncommon (Figure 3). Comments on usage appeared in 24% of responses on Homework 1 and only 2%–11% of total comments for other Homework assignments. Of the usage comments, the most common category (representing 51.2% of all usage comments), and the only usage category identified in all nine videos, was that students watched the video multiple times. Technical issues were reported in eight of nine videos (all except HW7 Origin of Birds) and were much more common in the first video (6.5% of total comments) than in subsequent videos (0%–1.1% of total comments). Reported technical issues included difficulty with audio and video streaming (i.e., “glitching”), as well as issues with accessing videos through the course LMS. Comments on altering video speed were uncommon (0%–3% of total comments), though students were more likely to watch videos at faster speeds than slower speeds. Students also reported using or desiring captions and transcripts for seven out of the nine videos.

#### 3.1.2 Comments on video length

**FIGURE 4:**
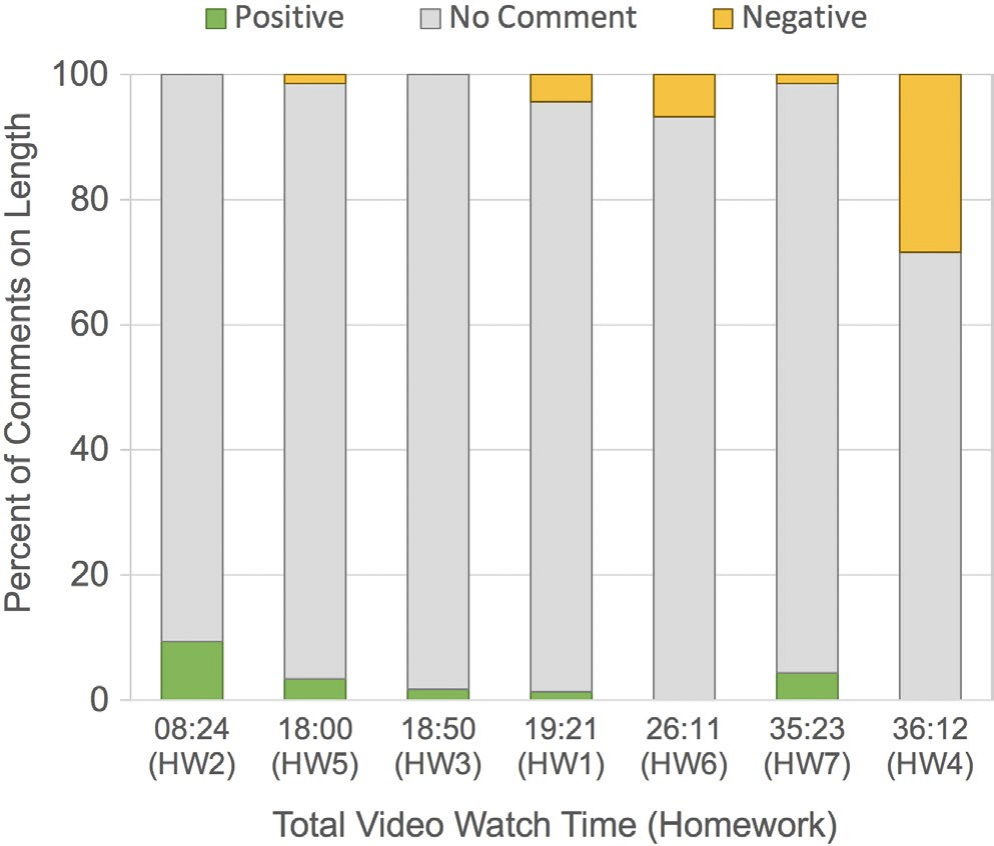
Summary of student comments on video length. Percent of student comments from individual homework feedback that were positive (green), negative (orange), or did not comment (gray) on length. *Note that the x‐axis is rank ordered by total video watch time, not homework assignment number*

Individual videos varied in length from 8:24 min to 36:12 min and averaged approximately 18:02 min. Two homework assignments incorporated multiple videos, so the average watch time per homework assignment was approximately 23:11 min. Across all homeworks, 9.4% of student comments mentioned video length. Of these comments, 62.6% found the videos too long and the remaining 37.4% found the videos of appropriate length. No homework assignment received zero comments on video length. The homework assignment with the longest watch time received the largest percentage of negative comments (28.4%) and no positive comments on length, while the homework assignment with the shortest watch time received the largest percentage of positive comments (9.3%) and no negative comments on length (Figure 4).

#### 3.1.3 Comments on homework questions

**FIGURE 5:**
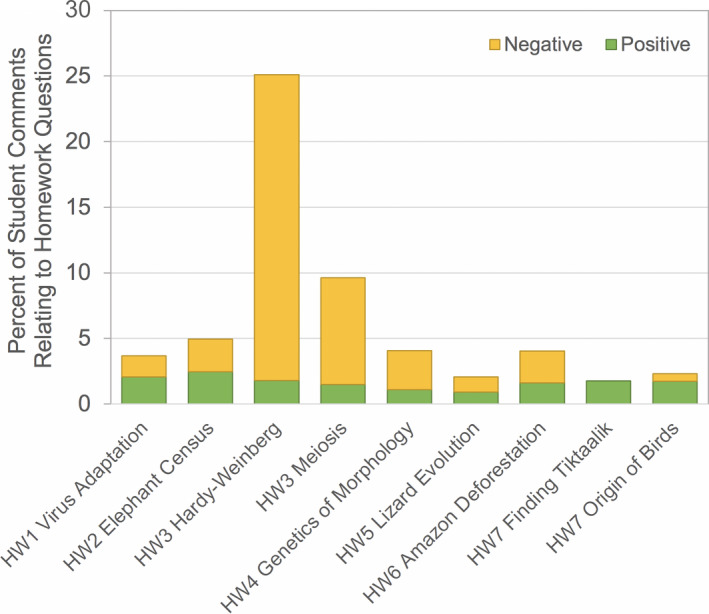
Number of positive (green) and negative (orange) comments about homework questions for each video

All videos received both positive and negative comments on the associated homework questions, with the exception of Finding *Tiktaalik* (HW7) which received only positive comments. The frequency of positive comments related to homework questions varied little across videos and were generally uncommon (0.9%–2.5% of total comments). With one exception, negative comments relating to homework questions were also uncommon (0%–8% of total comments). The exception was the Hardy–Weinberg equilibrium video (HW3), which received 52 negative comments on the homework questions, accounting for 23.3% of total comments for HW3 and 52.5% of all negative homework comments across all assignments (Figure 5).

#### 3.1.4 Comments on taxonomic group or region of study

**FIGURE 6:**
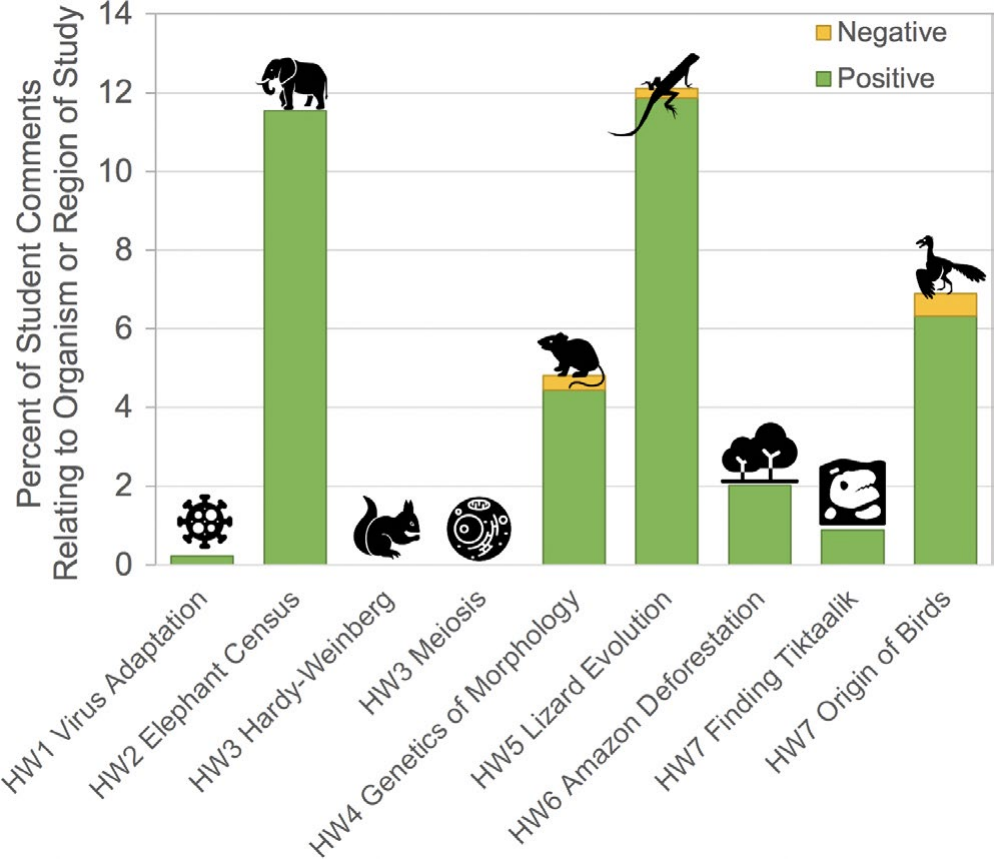
Percent of student responses that contained positive (green) or negative (orange) comments on the organism or habitat covered in each video. Icons on each bar represent the organism or habitat covered in the respective video (icon source: the Noun Project)

With one exception, all videos included in this study focused on a particular organism or habitat (the exception being the Meiosis video in HW3, which focused on division of individual cells within a multicellular organism). Interestingly, with one exception, all videos that focused on a particular taxon or habitat of study received positive comments specifically about the taxon or region (Figure 6, 0.2%–11.9% of total comments). The one exception was the Hardy–Weinberg video (HW3), which followed the population genetics of a fictitious population of hand‐drawn squirrels. Three taxonomic groups received negative comments (0.2%–0.6% of total comments).

#### 3.1.5 Comments on video and presenter style

**FIGURE 7:**
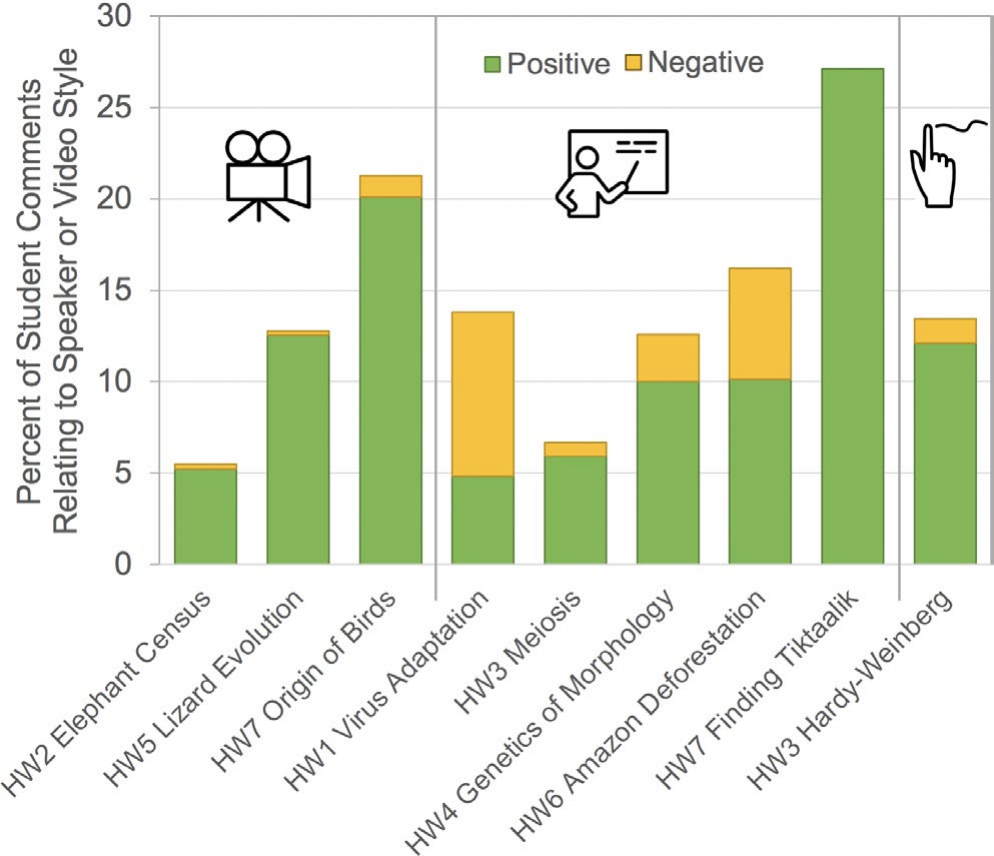
Percent of student responses for each video that contained positive (green) or negative (orange) comments on the speaker or video style. Videos are grouped by overall production style: documentary (left; camera icon), lecture (middle; presenter icon), and whiteboard drawing (right; drawing hand icon)

**FIGURE 8:**
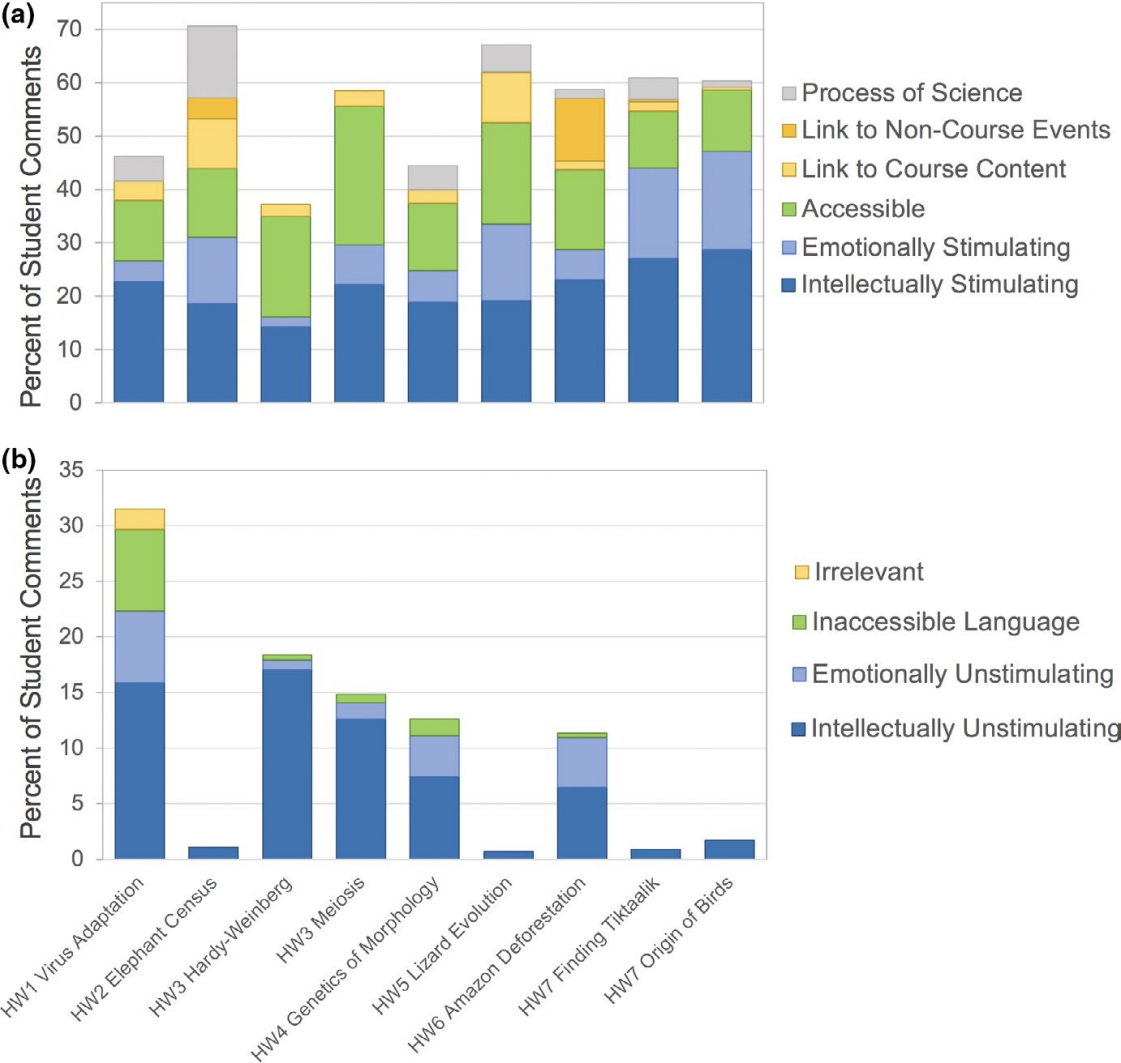
Summary of positive (a) and negative (b) student comments related to homework learning goals. See Table 3 for descriptions of each category. *Note the difference in y‐axis scale between the two panels*

All videos received positive comments specific to the presenter or the style of the video, and all but one also received negative comments (Figure 7). Generally speaking, documentary‐style videos received a higher ratio of positive to negative comments about video or speaker style (Figure 7) with positive comments appearing in 5%–10% of responses and negative comments appearing in <1.2%. In lecture‐style videos, positive comments appeared in 4%–27% of responses and negative comments appeared in 0%–9% (Figure 7). Documentary‐style videos also consistently received few comments indicating that they were intellectually unstimulating (0.7%–1.7% of total comments), and no comments indicating that they were emotionally unstimulating (Figure 8b). One lecture‐style video exhibited similar results (Figure 8). This video, Finding *Tiktaalik* (HW7), received the highest percentage of positive comments on video or speaker style (27%) and also was the only video of any format to receive no negative comments on style (Figure 7). Like the documentary‐style videos, Finding *Tiktaalik* also received few negative comments regarding engagement (0.89%, Figure 8b).

#### 3.1.6 Comments related to assignment goals

All videos received comments indicating that students found them intellectually stimulating (14.3%–28.7%), emotionally stimulating (1.8%–18.4%), accessible (10.7%–25.9%), and relevant to course content (0.6%–9.4%, Figure 8a). In seven of the nine videos, students commented positively on the process of science represented in the video (1.6%–13.5%). Additionally, in comments for two videos (HW2 Elephant Census and HW6 Amazon Deforestation), students made connections between the research highlighted in the video and noncourse events or global issues (3.8%–11.7%). The frequency of assignment goal‐related comments varied widely across videos and ranged in total from 37.2% to 70.6% of total comments (Figure 8a).

Negative comments related to assignment goals were generally less common (Figure 8b), ranging from 0.7% to 31.5% of total comments. All videos received comments indicating students found the video intellectually unstimulating (0.7%–17%), and five videos received comments indicating students found the video emotionally unstimulating (0.9%–17%) and inaccessible due to terminology and jargon (0.4%–7.4%). Only one video (HW1 Virus Adaptation) received comments that students found it irrelevant (i.e., they were unable to identify how the video connected to course concepts or other content; 1.8%).

### 3.2 Student feedback from end‐of‐quarter evaluations

**FIGURE 9:**
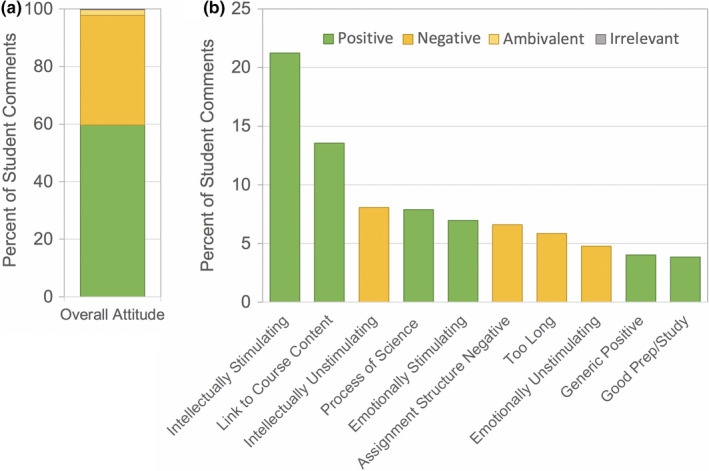
Summary of end‐of‐quarter student feedback on video homework assignments overall. (a), percent of student comments that were positive (green), negative (orange), ambivalent (yellow), or irrelevant (gray); (b), the 10 most common categories identified in end‐of‐quarter feedback

Approximately 60% of the anonymous comments from the end‐of‐quarter evaluations were positive (Figure 9). The top 10 most common responses included 6 positive and 4 negative categories. Interestingly, common positive and negative categories were occasionally opposing perspectives. For instance, “Intellectually Stimulating” and “Intellectually Unstimulating” were both in the top 10, as were “Emotionally Stimulating” and “Emotionally Unstimulating.”

## 4 DISCUSSION

### 4.1 Does length matter?

Previous research has suggested that optimal video length for online activities should fall in the 6–9 min range (Guo et al., 2014; Risko et al., 2012). Only one of the videos selected for this class was this short and the longest video was four times this recommended length. Comments on length tended to be uncommon in the feedback on each individual video (Figure 4), but the comment that homework assignments generally were too lengthy and time‐consuming was in the top 10 responses on the end‐of‐quarter feedback (Figure 9). It is possible that the end‐of‐quarter surveys are a more accurate reflection of student perceptions given their anonymity, although even in this format, complaints about length were relatively infrequent overall (Figure 9).

To illustrate the complexity around students’ perspectives on length, consider Homeworks 3 and 7, compared to other homeworks of similar length. Homework 3 included two videos that totaled nearly 19 min and which received a relatively high proportion of comments indicating that they were intellectually unstimulating (Figure 8b). Yet this assignment received no negative, and 1.7% positive, comments about length (Figure 4). Homework videos of similar total watch time (HW5 at 18:00 min and HW 1 at 19:21 min) received a similar proportion of positive comments (1.3%–3.4%) and also 1.5%–4.4% negative comments about length (Figure 4). Similarly, Homeworks 4 and 7 had nearly identical total video watch time, yet received disparate comments regarding length (Figure 4). Specifically, Homework 4 received the most negative comments on length of any video (*n* = 46, Figure 4), as would be predicted by its longest run time and by the lack of engagement reported by students (Figure 8b). Yet Homework 7, which was only 49 s shorter than Homework 4, received not only far fewer negative comments on length (*n* = 2), but also some positive comments (*n* = 6, Figure 4). These comparisons suggest that multiple short videos were more palatable to the students than single videos of the same total length.

### 4.2 Writing good(?) questions around videos

Including questions with videos is recommended to shift videos away from a passive and toward an active learning experience (Brame, 2016). Accordingly, we found that appreciation of the added prep/study opportunity was one of the top positive categories of student comments on the video homework assignments overall (Figure 9). Questions associated with the homework assignments were focused primarily around the process of science and linking concepts discussed in the video with other course topics. Common question styles included interpretation of a data figure shown in the video (or found in related publications), evaluation of a hypothesis proposed in the researcher's study, explanation of an outcome reported in the study, and consideration of which related course topics were illustrated or applicable to the video content (though not explicitly stated in the video). Homework 3 received 5–15 times more negative comments about the homework questions than any other assignment (Figure 5). This homework assignment was the only one that required calculations (related to Hardy–Weinberg equilibrium) and also went the furthest beyond just the content presented in the video, asking students to extrapolate the concepts in the video to a fictitious scenario without a worked example. Interestingly, this assignment received a similar percentage of *positive* comments about the homework questions as other assignments (Figure 5).

In an introductory course of over 300 students, preparedness and background knowledge on course concepts vary considerably between students. Homework 3 contained 7 questions which varied in difficulty and included two challenging questions to prepare students for possible examination questions. Just two challenging questions led to extreme student frustration with the assignment, which in retrospect, could have been alleviated by either explicitly framing the difficulty for the students before hand (see Importance of Framing section, below) and/or providing a small hint within the assignment to put students on the correct logical “path.”

### 4.3 Student access and technical issues

With the exception of HW1, few comments reported technical issues with accessing the videos and associated homework questions (Figure 3). This likely reflects the learning curve on a new style of assignment, as well as the fact that the first video (unlike the others) only required the students to watch the second half, which was confusing for many students to know where to begin the video. All videos had captions and/or transcripts available, though several students requested captions on the first video, apparently unaware of how to access them. Consequently, the instructor showed the students in class how to access captions on YouTube videos and these responses were largely reduced (though not fully eliminated) in subsequent videos.

All videos received comments that students watched the video several times (Figure 3). These comments were most numerous in videos also deemed by students to be less intellectually and emotionally stimulating (HW1, HW3, HW4, and HW6; Figure 8b) and possibly reflect students rewatching sections of the video that related to the homework questions. Use of captions and transcripts was also higher in videos for Homeworks 1 and 4 for presumably the same reason. Interestingly, a small number of students noted that they watched the videos from Homework 7 (Finding *Tiktaalik* and The Origin of Birds) multiple times for their own enjoyment and to share with friends or relatives. These comments were supported by the high intellectual and emotional engagement that students reported for these videos (Figure 8a). Therefore, comments on usage could reflect both positive and negative responses to videos.

### 4.4 What video styles do students find most engaging?

The primary purpose of requesting feedback from students on each video was to gain an understanding of what components of the videos were most important to the students. Not surprisingly, intellectual and emotional engagement with the topic, accessibility of content, and narration by a charismatic speaker or engaging video style (e.g., documentary format) were the most prevalent positive video traits identified by students (Figure 2). These comments are consistent with evidence‐based suggestions that videos should reduce cognitive load and promote student engagement for highest learning success (Brame, 2016). Beyond these top traits, our results indicate that there are multiple ways in which students respond positively to videos.

Unsurprisingly, documentary‐style videos generally received few comments indicating that they were intellectually or emotionally unstimulating (Figure 8b) and also received a relatively higher percentage of positive comments regarding the taxonomic group of study (Figure 6, though it should be noted that a positive taxon effect could be reversed if the video highlights an organism commonly feared by students, e.g., spiders). Interestingly, The Great Elephant Census (HW2) received relatively fewer positive style‐related comments than the other documentary‐style videos (Figure 7). Yet, it received a high percentage of positive comments related to taxonomic group (Figure 6) and received the highest cumulative proportion of comments relating to course learning goals (Figure 8a). This video highlighted transect and quadrat sampling methods, which the students had used themselves in laboratory activities the week prior, and student comments identified this connection. Overall, the high student engagement with documentary‐style videos is consistent with increased learning gains for this video style reported elsewhere (Boy et al., 2020; see data on “Narrative Explanatory Film” category).

Our findings indicate that lecture‐style or animation videos can offer valuable learning opportunities, even if they are viewed as generally less engaging than documentary‐style videos. Indeed, the lecture‐style video Finding *Tiktaalik* received similar comments to the documentary‐style videos, with the exception of taxonomic group interest. Also, the Amazon Deforestation video received the most comments on links to noncourse events (Figure 8a), as students were aware of the wildfires in the Amazon during 2019 and were interested to learn more about this region. Additionally, despite comments indicating lack of intellectual and emotional engagement (Figure 8b), the two videos focused on refreshing students’ understanding of course topics in Homework 3 received a high percentage of positive comments about accessibility (Figure 8a). Notably, students reported familiarity with Bozeman Science and some already used this YouTube channel on their own for studying.

### 4.5 Summary

In summary, students responded positively to videos that incorporated at least one of the following: documentary‐style filming, very clear links to course content (especially hands‐on activities completed by the students), relevance to recent world events, clarity on difficult topics, and/or charismatic narrators or species. In addition to enjoying the “feel” and engaging style of documentary‐type videos, students responded positively to seeing the scientists “at work” gathering data in the field and seeing organisms of study in the wild. These components could be incorporated into lecture‐style videos via photographs or embedded videos of fieldwork and study organisms. Our analysis also found that students are amenable to multiple videos of shorter length.

### 4.6 Instructor tips

Below, we outline instructor strategies and resources for integrating videos into their teaching, based on the findings above and our collective professional experience.

#### 4.6.1 The importance of framing

It is important for educators to promote student engagement with videos by clearly stating why the material was selected for a given course with the given students in mind (Brame, 2016). As the instructor, LMG made a conscious effort to frame the homework assignments for the students throughout the quarter. Prior to students completing the first homework assignment, this framing included the following:

● Explaining the purpose of the video homework in terms of grading structure (low‐stakes formative assessments)
● Explaining the purpose of the video homework in terms of learning (supplementing flipped discussion sections, showcasing current research by current scientists)
● Explaining the purpose of the voluntary feedback question
● Explicitly encouraging honest feedback, and instructor openness to negative perspectives

Continued framing throughout the quarter included (italicized bullet points represent framing components that the instructor would, in the future, include prior to the first homework assignment):

● *Showing students how to access captions and transcripts for videos*
● *Encouraging students to look up unfamiliar terminology in their textbook or online*
● Encouraging honest feedback by showing anonymized student comments about videos to highlight different student perspectives on the assignment, explicitly including a variety of negative and positive comments and stating that both are useful for the instructor. As student feedback declined throughout the quarter, this strategy was repeated halfway through the course and student responses increased. This approach also served to normalize the experience for students who struggled with the material.
● Explicitly referencing video and homework content during lecture and discussion
● Continuing to clarify instructor perspective on the videos. For instance, the instructor considered Homework 4 to be the most difficult video and notified students ahead of time that they may need to allocate more time that week to the homework assignment.
● Responding to student frustration on difficult homeworks. Following Homework 3, the instructor provided a worked example of the difficult questions, included extra practice on this topic on the examination study guides, and reminded students that one purpose of homework assignments is to give students practice on difficult concepts when the grade point stakes are low, in order to better prepare them for high‐stakes examinations.
● Explicitly encouraging honest feedback on the end‐of‐quarter evaluations, including instructor openness to negative perspectives, and ensuring that students understood their responses were entirely anonymous and not visible to the instructor until after final course grades were submitted to the registrar.

#### 4.6.2 Additional resources

Many undergraduate biology educators will continue remote teaching into the 2021–2022 academic year, and many will be searching for free online videos to supplement their course material. This case study was performed on video homework assignments as a component of a traditional in‐person course. The importance of choosing engaging videos is even higher under scenarios of fully remote or hybrid instruction. In addition to the resources described in this case study (Bozeman Science, HHMI BioInteractive, and iBiology), we recommend educators search for supplemental videos in other well‐known resources including Ted Ed, Kahn Academy, JOVE, and Crash Course. Although not described in this case study, it may be useful to make associated questions interactive by embedding them within videos (Brame, 2016). There are several resources that allow educators to interpolate questions throughout videos, including EdPuzzle, PlayPosit, Camtasia, and Nearpod.

## CONFLICT OF INTEREST

Brittany N. Anderton is an employee of iBiology, a nonprofit, open‐access science education organization.

## AUTHORS CONTRIBUTION

**Laci M. Gerhart:** Conceptualization (equal); Data curation (lead); Formal analysis (lead); Investigation (lead); Methodology (lead); Project administration (lead); Supervision (lead); Visualization (lead); Writing‐original draft (equal); Writing‐review & editing (lead). **Brittany N. Anderton:** Conceptualization (equal); Funding acquisition (lead); Methodology (supporting); Supervision (supporting); Visualization (supporting); Writing‐original draft (equal); Writing‐review & editing (supporting).

## Data Availability

To ensure FERPA (Family Educational Rights and Privacy Act) compliance, the authors will share only aggregated, deidentified data upon request.
